# Supplementary material for: Causal interplay between lactose intolerance and gut microbiota: a combined bidirectional Mendelian randomization and in vivo validation study
Source: Front Nutr. 2026 Jun 1;13:1803337. doi: 10.3389/fnut.2026.1803337 (PMC13265576; doi:10.3389/fnut.2026.1803337)
Supplement: Supplementary file 1 [file Data_Sheet_1.zip › supplementary materials/Forward/leave one out cross validation/ebi-a-GCST90027564.finngen_R12_E4_LACTONAS.pdf]

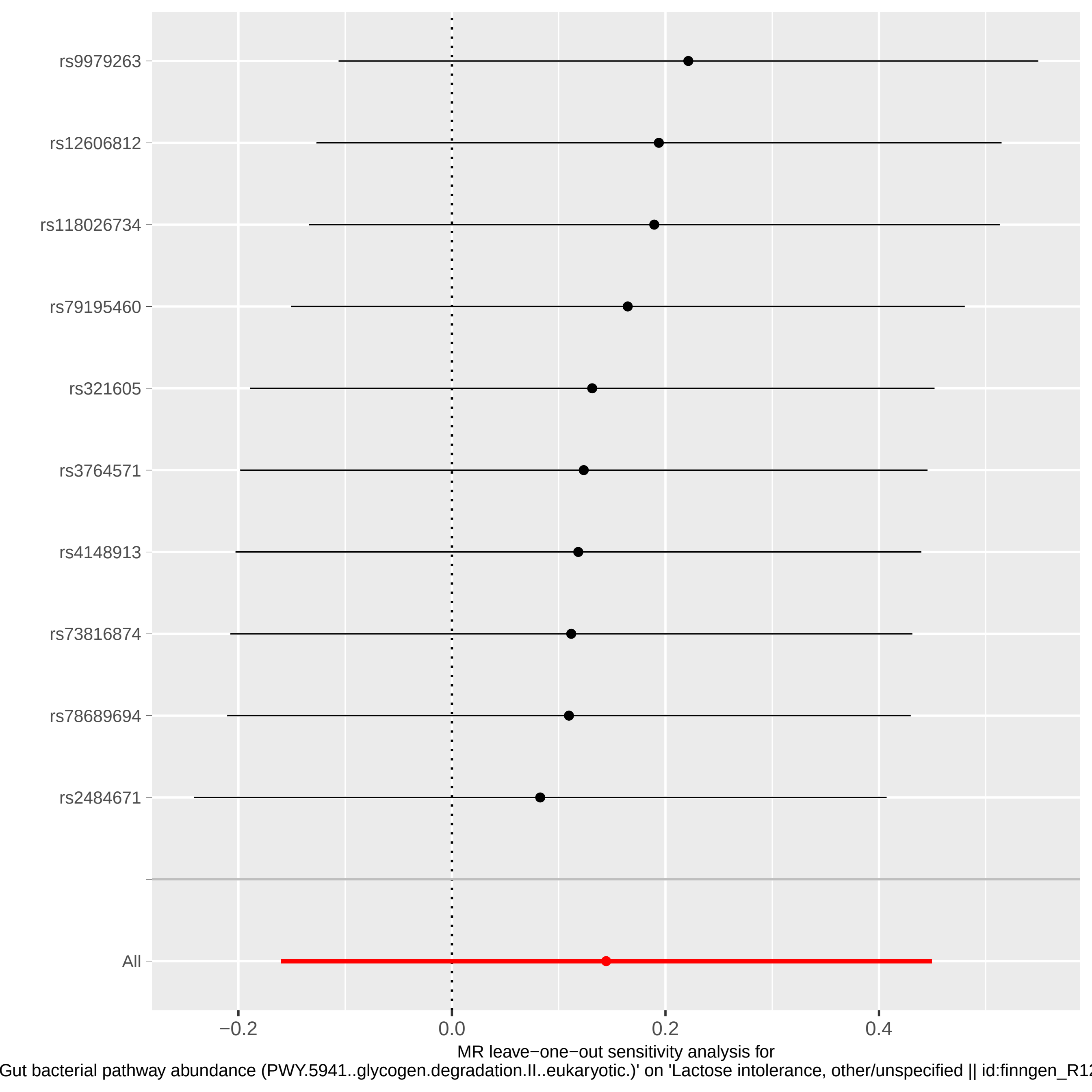
rs9979263
rs12606812
rs118026734
rs79195460
rs321605
rs3764571
rs4148913
rs73816874
rs78689694
rs2484671
All
−0.2
0.0
0.2
0.4
MR leave−one−out sensitivity analysis for
Gut bacterial pathway abundance (PWY.5941..glycogen.degradation.II..eukaryotic.)' on 'Lactose intolerance, other/unspecified || id:finngen_R1
